# Macrophages in Chronic Liver Failure: Diversity, Plasticity and Therapeutic Targeting

**DOI:** 10.3389/fimmu.2021.661182

**Published:** 2021-04-02

**Authors:** Arjuna Singanayagam, Evangelos Triantafyllou

**Affiliations:** ^1^ Infection and Immunity Clinical Academic Group, St. George’s University Hospitals NHS Foundation Trust, London, United Kingdom; ^2^ Section of Hepatology and Gastroenterology, Department of Metabolism, Digestion and Reproduction, Imperial College London, London, United Kingdom

**Keywords:** cirrhosis, macrophages, liver injury, liver inflammation, liver fibrosis, chronic liver failure, Kupffer cells

## Abstract

Chronic liver injury results in immune-driven progressive fibrosis, with risk of cirrhosis development and impact on morbidity and mortality. Persistent liver cell damage and death causes immune cell activation and inflammation. Patients with advanced cirrhosis additionally experience pathological bacterial translocation, exposure to microbial products and chronic engagement of the immune system. Bacterial infections have a high incidence in cirrhosis, with spontaneous bacterial peritonitis being the most common, while the subsequent systemic inflammation, organ failure and immune dysregulation increase the mortality risk. Tissue-resident and recruited macrophages play a central part in the development of inflammation and fibrosis progression. In the liver, adipose tissue, peritoneum and intestines, diverse macrophage populations exhibit great phenotypic and functional plasticity determined by their ontogeny, epigenetic programming and local microenvironment. These changes can, at different times, promote or ameliorate disease states and therefore represent potential targets for macrophage-directed therapies. In this review, we discuss the evidence for macrophage phenotypic and functional alterations in tissue compartments during the development and progression of chronic liver failure in different aetiologies and highlight the potential of macrophage modulation as a therapeutic strategy for liver disease.

## Introduction

Liver disease is a global health burden with recent estimates suggesting that 844 million people worldwide have chronic liver disease (CLD), with a mortality rate of 2 million deaths per year: approximately, 1 million due to complications of cirrhosis and 1 million due to viral hepatitis and hepatocellular carcinoma (HCC) (1, 2). Chronic liver injury, most commonly caused by alcohol, infection or liver fat accumulation associated with features of the metabolic syndrome, triggers the activation of liver-resident and infiltrating immune cells, resulting in inflammation, progressive fibrosis, disrupted architecture, vascular changes and aberrant regeneration, which are defining characteristics of liver cirrhosis (2). Management of patients with cirrhosis is limited to treating the underlying cause and, where appropriate, liver transplantation. However, the latter is difficult to access in much of the world where donor organ supply is insufficient to meet demands (2). Therefore, developing new effective therapies for CLD patients would likely have a considerable benefit on morbidity and mortality. This review focuses on the diverse and dynamic role of macrophages in CLD and how their modulation might offer novel therapeutics.

## Chronic Liver Failure

### Cirrhosis

**Figure 1:**
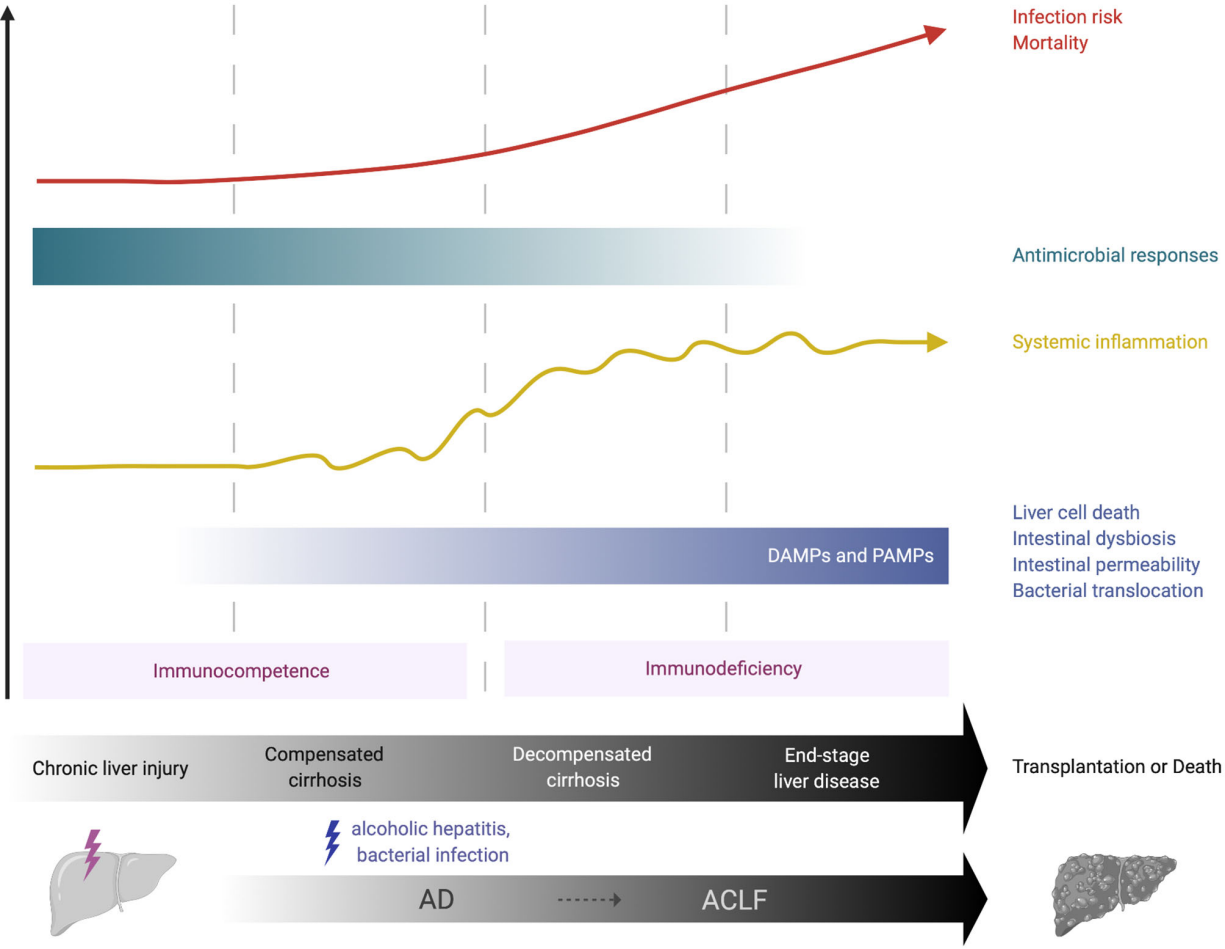
Immunological features of chronic liver failure. Over the course of compensated or decompensated cirrhosis, an acute precipitating event such as alcoholic hepatitis or bacterial infection may challenge liver homeostasis and lead to acute-on-chronic liver failure (ACLF). In compensated cirrhosis, liver-derived DAMPs, for example from necrotic hepatocytes, may activate the local immune system and initiate systemic inflammation. In decompensated cirrhosis, increasing alterations in intestinal homeostasis (e.g., bacterial overgrowth, increased permeability) allow the pathological translocation of bacteria, bacterial products (e.g., LPS) and PAMPs into the liver, thus providing further chronic stimulation of innate immune cells and propagation of local and systemic inflammation. This may be crucial in the transition from the immunocompetent state (pro-inflammatory immune response) to a more hyporesponsive, immunodeficient state that is observed in advanced cirrhosis. The latter increases the risk of life-threatening infections and substantially contributes to the high morbidity and mortality observed in these patients. ACLF, acute-on-chronic liver failure; AD, acute decompensation; DAMP, damage-associated molecular pattern; PAMP, pathogen-associated molecular pattern.

Cirrhosis comprises two consecutive but potentially reversible stages: compensated (asymptomatic) and decompensated cirrhosis (3, 4). The term acute decompensation (AD) of cirrhosis defines the acute development of one or more major complication(s) in patients (5). These complications include ascites, hepatic encephalopathy and variceal haemorrhage. The first AD episode marks the transition from the compensated to the decompensated stage. During AD, patients are extremely prone to develop bacterial infections, to the point that bacterial infections have been considered as the fourth major complication of the disease (5). Cirrhosis features a gradually dysfunctional immune response that encompasses systemic inflammation, which can exacerbate clinical manifestations of cirrhosis (e.g., hemodynamic derangement and kidney injury) and, as disease progresses, immunodeficiency and impaired antimicrobial functions that are associated with high infection risk (**Figure 1**). The term cirrhosis-associated immune dysfunction (CAID) defines the dynamic spectrum of the immunological perturbations that develop in these patients [reviewed in (6–8)].

Over the course of compensated or decompensated cirrhosis, an acute precipitating event, most commonly bacterial infections and acute alcoholic hepatitis, challenge liver homeostasis and may lead to a syndrome called acute-on-chronic liver failure (ACLF) (9, 10). ACLF is defined by acute decompensation of cirrhosis, hepatic and/or extra-hepatic organ failure with high short-term (28-day) mortality (3, 4). Inadequate immune responses to the precipitating event are key to its pathogenesis, with high grade of systemic/local inflammation and immunodeficiency, that lead to further organ failure. This is profound in the setting of ACLF, which resembles the immunopathology of sepsis, with an initial systemic inflammatory response (cytokine storm) followed by a compensatory anti-inflammatory response that can impair immune defence against infections (3, 4).

### Burden of Infections in Cirrhosis

Patients with cirrhosis develop a range of complications, with infections being one of the most clinically important issues, associated with high morbidity and mortality. Bacterial infections have been shown to occur frequently (32-34%) in patients with advanced cirrhosis; in 30–50%, infection is the cause of hospital admission, and a further 15–35% develop nosocomial infections (as compared to 5-7% of general population) (11). Infections can further trigger hepatic decompensation and are well-known precipitants for encephalopathy, hepatorenal syndrome (HRS) and development of ACLF (5). Most recent data derive from the CANONIC and PREDICT studies, that were observational prospective investigations in large cohorts of non-selected patients hospitalised with AD. Among 407 patients with AD-ACLF, the incidence of infections at hospital admission and during a 28-day follow-up period was 65% (12). The corresponding incidence of infections in 1,071 patients with AD-no-ACLF was 53% (9, 13). Globally, the most common infections in cirrhotic patients are spontaneous bacterial peritonitis (SBP) (27%), urinary tract infections (22%), pneumonia (19%), spontaneous bacteraemia (8%), skin and soft tissue infections (8%) (14). The majority of infections identified in cirrhotic patients are caused by Gram-negative bacteria (e.g., *Escherichia coli*, *Klebsiella Pneumoniae*) of intestinal origin; Gram-positive bacterial infections are less frequent (e.g., *Staphylococcus aureus*) (14, 15). The epidemiology of infections in cirrhosis continuously change, with recent studies demonstrating increasing prevalence of multidrug resistant organisms (29% to 38% increase from 2011 to 2017-2018) (14, 15).

### Systemic Inflammation and Immune Dysfunction in Cirrhosis

Innate immune cells (mainly neutrophils, monocytes and macrophages) are primed to detect tissue damaging or infectious insults, and therefore are key orchestrators of inflammatory responses. During progression of CLD these cells initiate and drive both liver and systemic inflammation by recognising/responding to damage-associated molecular patterns (DAMPs) released from injured/activated liver cells and/or pathogen-associated molecular patterns (PAMPs) (16). Fibrosis occurs following chronic liver injury from an insult (toxic, metabolic, or infectious) which can perpetuate inflammation. Fibrogenesis is the common pathological mechanism that causes cirrhosis; it is a complex and dynamic process that involves an array of activated resident or recruited inflammatory cells (e.g., platelets, macrophages), hepatic stellate cells (HSCs), hepatocytes, other extracellular matrix (ECM) producing cells and extracellular signals [e.g., cytokines, chemokines, adipokines or reactive oxygen species (ROS)] (17–19). The activation of HSCs - transdifferentiation of quiescent cells into proliferative, fibrogenic myofibroblasts - is a well-established driver of fibrosis [reviewed in (17–19)]. This is a cardinal process in which quiescent HSCs responding to hepatic injury downregulate the expression of GFAP, peroxisome proliferator-activated (PPAR)-γ and vitamin A and become activated HSCs (18). Following stimulation with cytokines and fibrogenic signals, of which transforming growth factor (TGF)-β is the most potent, HSCs upregulate α-smooth muscle actin expression, increase transcription of collagen type I *via* SMAD-3 amongst other routes, and traffic to sites of injury where they secrete ECM resulting in fibrous scar formation (20). With fibrosis progression, cirrhosis may ultimately develop.

Advanced cirrhosis is closely associated with pathological bacterial translocation (BT), the increased rate of translocation of bacteria or bacterial products (e.g. lipopolysaccharide [LPS], bacterial DNA and peptidoglycans) from the gut to mesenteric lymph nodes and other tissues (21). SBP is considered to be the hallmark manifestation of pathological BT and is a multi-faceted process. This involves small bowel dysmotility, small intestinal bacterial overgrowth (SIBO), increased intestinal permeability and dysbiosis. The exact mechanisms of enhanced gut permeability are not yet fully understood; loosening of the intestinal epithelial tight junctions and reductions in luminal antimicrobial peptides, such as secretory component and mucins, may have a role (21, 22). Dysbiosis sees a decrease in the quantity of beneficial autochthonous bacteria (e.g., *Ruminococcacaea* and *Lachnospiracaea)* and an increase in potentially pathogenic taxa (e.g., *Enterobacteriaceae* and Bacteroidaceae), termed the cirrhosis dysbiosis ratio (23). Why such intestinal changes occur in cirrhosis is unclear but alcohol, portal hypertension and impaired local immune responses have been implicated (24). Impaired hepatic immune responses and portosystemic collaterals in advanced cirrhosis provide further opportunity for translocated products to pass from the intestinal lumen to the systemic circulation, chronically stimulating innate immune cells and enhancing systemic inflammation. This increase in bacterial products may be crucial in the switch from the initially pro-inflammatory immune response described in early cirrhosis to a more hyporesponsive, immunodeficient state observed in decompensated cirrhosis (**Figure 1**).

## Monocyte Dysfunction in Chronic Liver Failure

Circulating monocytes, a key component of the mononuclear phagocyte immune system, play pivotal roles in defence against infections and contribute to the systemic inflammation in chronic liver failure. In addition, they augment the local macrophage pool *via* their recruitment to inflammatory sites after a sterile/tissue-damaging or infectious insult to the liver (10, 16, 25). Human monocytes are divided into three major subsets: classical (CD14^+^CD16^−^), intermediate (CD14^+^CD16^+^) and non-classical (CD14^dim^CD16^+^). Each of these subsets can be distinguished from each other by their differential expression of surface markers and their distinct functions [for an up-to-date review see (26)]. Classical monocytes are comparable to the inflammatory Ly6C^+/high^ CCR2^high^ murine subset whilst non-classical monocytes resemble the patrolling Ly6C^-/low^ CX3CR1^high^ subset. The former cells rapidly infiltrate tissues in response to injury, have the capacity to differentiate into dendritic-like cells, and are primed for phagocytosis and innate immune sensing. Ly6C^low^ monocytes crawl along endothelial cells, coordinating the clearance of apoptotic cells and cellular debris (27). Similarly, non-classical human monocytes are suited to adhesion and Fc gamma-mediated phagocytosis. Whilst intermediate monocytes do not demonstrate such crawling behaviour, they are a heterogenous population adapted for antigen presentation, cytokine production and apoptosis regulation (26, 28–30). Interestingly, a study using deuterium labelling in humans has suggested that intermediate and non-classical monocytes emerge sequentially from the pool of classical monocytes (31).

The number of circulating monocytes increases in relation to the severity of cirrhosis (from Child-Pugh A to Child-Pugh C and AD) (32, 33). Of note, an expansion of intermediate monocytes has been described in the circulation and liver of patients with cirrhosis progression (32, 34). Dysregulation of circulating monocytes and their contribution to systemic immune paresis and the pathophysiology of cirrhosis have been well-documented [reviewed in (10, 35)]. The most consistent finding with regard to monocyte phenotype in chronic liver failure is reduced HLA-DR expression. This reduction, that likely compromises their antigen presentation capacity and the development of adaptive immunity, is considered a hallmark of “endotoxin tolerance”, the immunosuppressed state in which monocytes are refractory to further LPS stimulation or other microbial stimuli. Monocyte HLA-DR expression levels diminish progressively in relation to cirrhosis severity and are associated with adverse clinical outcomes (33, 34, 36–38). Furthermore, monocyte production of pro-inflammatory cytokines in response to microbial cues (e.g., LPS) is key to innate defences against infection as such cytokines can enhance their antimicrobial functions. Monocyte inflammatory (e.g., TNF-α, IL-6) cytokine secretion appears to diminish with advancing chronic liver disease particularly in AD and late stage ACLF (32–34, 36–39). Similarly, plasma derived from AD, but not compensated, cirrhosis suppresses LPS-stimulated TNF-α by healthy monocytes (40).

A role for the anti-inflammatory cytokine IL-10 in driving monocytes towards this “endotoxin tolerant state” has been proposed but this mechanism may involve a variety of pathogen-derived signals or cell-secreted cytokines and proteins (34–37, 41). Recently, the reduced HLA-DR expression on monocytic cells in advanced stages of cirrhosis (AD/ACLF) has been attributed to the expansion of monocytic (CD11b^+^CD15^-^CD14^+^HLA-DR^-^) myeloid-derived suppressor cells (M-MDSCs) in the circulation (38). M-MDSCs have great pathological significance in chronic liver failure as they exert highly immune-suppressive functions: they decrease T cell proliferation, produce low TNF-α and IL-6 levels after toll-like receptor (TLR) stimulation and have reduced bacterial (*E. coli*) phagocytosis capacity (38).

The TAM (TYRO3/AXL/MERTK) family of receptors, which are critical regulators of innate immune responses and promote clearance of apoptotic cells (termed efferocytosis), are shown to contribute to systemic immune paresis in liver failure (33, 42, 43). Brenig et al. recently identified a distinct immune-regulatory population of (CD14^+^HLA-DR^+^) AXL-expressing monocytes that is expanded in parallel with progression of cirrhosis prior to the AD/ACLF stage and correlates with development of infection and one-year mortality (33). This subset displayed attenuated TNF-α and IL-6 secretion following TLR stimulation, reduced T cell activation but had preserved bacterial phagocytosis and enhanced efferocytosis capacity (33). In patients with AD/ACLF, the emergence of a MERTK-expressing monocyte population is described to decrease innate immune responses to LPS and to associate with disease severity and adverse outcomes (34, 42). Proof-of-concept *in vitro* inhibition of either AXL or MERTK was shown to enhance monocyte inflammatory responses in ACLF samples. Therefore, TAM receptor targeting may be a new therapeutic strategy to restore monocyte function in cirrhosis. However, this needs further clinical evaluation (35).

## Tissue Macrophages in Chronic Liver Failure

### A. Liver Macrophages in Steady State

Macrophages are the most abundant hepatic immune cells with pivotal roles: from maintaining liver homeostasis and immune tolerance in the face of continuous exposure to harmless gut-derived antigens from food and commensal microbes or their products, to rapidly identifying pathogens and orchestrating immune responses for their elimination (16). In addition, they are functionally important for clearing cellular debris and metabolic waste, and regulating iron and cholesterol homeostasis (16). During steady state, the predominant macrophage population comprises resident Kupffer cells (KCs) which are capable of self-renewal and have a specific transcriptional program defined by their unique niche (44–46). KCs reside within sinusoidal blood vessels, in continuous contact with liver sinusoidal endothelial cells (LSECs), while they always extend a substantial fraction of their cell body into the perisinusoidal space of Disse where they can closely interact with HSCs and hepatocytes (47). This position of KCs is functionally important during liver homeostasis and injury. In the setting of inflammation, following a toxic, metabolic or infectious insult, the liver macrophage pool can further consist of recruited monocyte-derived macrophages (MoMFs) (10, 16). In mice, resident KCs can be identified by their specific (CD11b^int^F4/80^high^) MARCO^+^CLEC4F^+^TIM4^+^ expression profile while MoMFs are (CD11b^high^F4/80^int^) CX3CR1^+^ cells (45, 46, 48, 49). An additional population of liver capsular macrophages (LCMs) has been described to occupy the hepatic capsule. LCMs are CX3CR1^high^TIM4^-^ MoMFs, replenished at steady state by blood monocytes, that exert key immune surveillance and antimicrobial functions and therefore defend the liver from infections traversing the peritoneal cavity (50).

In humans, liver single cell RNA sequencing (scRNA-seq) studies have identified CD68^+^ CD163^+^MARCO^+^TIMD4^+^ resident KCs, CD68^+^MARCO^-^ recruited MoMFs and infiltrating CD14^+^ monocytes (51–53). Unbiased cross-species comparison suggests a highly conserved transcriptional signature among human and mouse KCs (53, 54). The dichotomous concept of M1/M2 polarisation cannot be reliably applied to hepatic macrophages, as with most tissue macrophages, since they simultaneously express M1/M2 markers and exhibit great plasticity which is dependent on their ontogeny, local and systemic environmental mediators and epigenetic programming. Liver macrophage ontogeny and heterogeneity is extensively covered elsewhere (16, 44, 55, 56).

### B. Liver Macrophages in Injury and Fibrosis

Tissue damage and hepatocyte injury generate coordinated wound healing responses that aim to restore healthy hepatic parenchyma. Release of DAMPs, ROS and changes in hepatocyte gene expression are triggered, promoting an immune response, clearance of apoptotic or dead cells, hepatocyte proliferation, matrix remodelling, and angiogenesis (57). Such changes result in increased expression of profibrogenic TGF-β, hedgehog signalling ligands and CXCL10 amongst others (58–61). If the insult abates, then resolution responses, including macrophage efferocytosis, restore tissue homeostasis. However, chronic injury eventually leads to dysregulated tissue repair and development of liver fibrosis. This is a dynamic process in which tissue damage and inflammation result in activation of HSCs, their epigenetic reprogramming and trans-differentiation into activated myofibroblast-like cells, cytokine and chemokine release, thus contributing to a profibrogenic microenvironment with subsequent ECM accumulation (mainly type I collagen) that limits parenchymal regeneration (19, 62). Early fibrosis can regress with injury removal and the activity of matrix degradation and remodelling by several matrix metalloproteinases (MMPs), myofibroblast senescence and HSC apoptosis (63, 64). MMPs are counteracted by tissue inhibitors of MMPs (TIMPs) and the balance of activity favouring TIMPs is associated with advancing fibrosis (64, 65).

Chronic liver injury eventually leads to progressive liver fibrosis and the development of cirrhosis with formation of fibrous septa, structural and vascular changes and regenerative nodules (58). Macrophage adaption during liver injury can depend on the nature of the insult (e.g., toxic, metabolic or infectious). In alcohol-induced liver damage, the number of hepatic macrophages increases *via* MoMF recruitment (66). Both KCs and MoMFs exhibit substantial plasticity with phenotypic and functional alterations depending on microenvironmental signals. Alcohol sensitizes KCs to TLR4-signalling and incites oxidative stress, which promotes an M1-like phenotype and LPS-induced cytokine production, particularly TNF-α, IL-6, IL-1β and CCL2 (67). In murine models of alcohol steatohepatitis, MoMFs are recruited to the liver in a NOTCH1-mediated mechanism with subsequent M1-like macrophage activation (68, 69). This process is compounded by alcohol-induced intestinal gut microbial dysbiosis, reduced gut epithelial integrity and subsequent heightened translocation of gut-derived microbial products including LPS (70). Chronic excess alcohol also results in hepatocyte fat accumulation (termed steatosis) and increased hepatocyte programmed cell death *via* apoptosis, necrosis, pyroptosis and ferroptosis, thus promoting further inflammation and injury (71). There are protective mechanisms at play including liver macrophage autophagy (an anti-inflammatory homeostatic intracellular pathway directing damaged organelles or cytosolic macromolecules to lysosomes for degradation), in which IRF-1 degradation, mitophagy (clearance of damaged mitochondria) and downregulation of inflammasome-dependent and independent pathways occur (72–74).

The multifactorial pathophysiology of alcoholic steatohepatitis overlaps significantly with non-alcoholic fatty liver disease (NAFLD) and its inflammatory and often progressive subtype non-alcoholic steatohepatitis (NASH). Macrophage activation occurs in response to endotoxins and translocated bacteria due to increased intestinal permeability, factors released from damaged or lipoapoptotic hepatocytes, as well as alterations in the gut microbiota and nutritional components (75). Collectively, these lead to a chronic inflammatory state resulting in disease progression. In obesity-associated NAFLD, release of free fatty acids from white adipose tissue promotes hepatocyte triglyceride synthesis and storage, and lipotoxicity with production of TNF-α, IL-6, IL-1β, IL-17A and macrophage-recruiting chemokines (e.g., CCL2, CCL5 and CXCL10). Macrophages in this condition have been studied in both human and disease models. Increased numbers of CD68+ macrophages are found in biopsies of young patients with more severe NAFLD (76), while in children with NAFLD numerous activated macrophages are located in the spaces between damaged hepatocytes (77). The portal infiltration of CCR2^+^ macrophages (78) appears to be an early event in human NAFLD, occurring already at the stage of steatosis before inflammation or fibrosis develops, but predicting progressive disease (79). In line with these data, lipogranuloma and macrophage alignment around steatotic hepatocytes have been observed (termed hepatic crown-like structures) (80).

Similarly, macrophage infiltration has been demonstrated in murine dietary models [e.g., high-fat diet (HFD), methionine–choline-deficient (MCD) diet] which have different strengths and limitations [reviewed in (75, 81)]. Undoubtedly, liver macrophages play a major role in the pathogenesis of NAFLD/NASH (75). Experimental evidence for this has come from studies in which depleting macrophages using genetic (LysM, myeloid-specific) models and clodronate liposomes protected mice from steatosis, liver damage and inflammation (82–85). Moreover, genetic deficiency and pharmacological inhibition of CCR2 decrease monocyte recruitment into the liver and ameliorate NASH in mice (78, 86). The level of macrophage heterogeneity in NASH, however, is greater than we initially thought. Recent scRNA-seq murine studies have shed more light into this, consistently demonstrating that resident KCs are lost during NASH progression and recruited monocytes enter the liver where they respond to niche-specific and inflammatory cues to become monocyte-derived KCs (MoKCs) or temporary MoMFs (49, 87–89). The distinct roles in disease pathology, functions and interactions of these macrophage subpopulations require more exploration, as further discussed below. For an overview of the immunometabolic interplay of hepatic macrophages and the adipose tissue-fatty liver crosstalk in NAFLD/NASH see also references (75, 90–92).

In both a mouse model of hepatitis B virus (HBV)-induced liver inflammation and in patients with viral-related chronic liver failure, Tan-Garcia et al. describe an intrahepatic infiltration of a population of pro-inflammatory CD14^+^HLA-DR^high^CD206^+^ myeloid cells (93). Likewise, Ohtsuki et al. observed an increase in the CD11b^+^F4/80^+^CD206^+^ intrahepatic macrophage population (labelled as “M2”) in HCV-infected transgenic mice, also producing more TNF-α and IL-6 following *ex vivo* LPS stimulation, when compared with M1 (CD11b^+^F4/80^+^CD11c^+^) (94). Chronic alcohol feeding of wild-type mice resulted in an increased frequency of CD206^+^ CD163^+^ M2 macrophages but also increased expression of M1 genes (*TNF-α*, *MCP1* and *IL-1β*) and M2 genes (*Arg1*, *Mrc1* and *IL-10*), with increased expression of Kruppel-like factor 4 (KLF4) promoting an M2 phenotype (95). Gut bacterial translocation appears important in viral chronic liver injury; interestingly, HBV-infected mice treated with oral antibiotics showed depleted hepatic CD14^+^CD206^+^ populations (93). Corroborating this, enhanced gut bacterial translocation has been noted in patients from HBV and HCV infection (96). This highlights the shared mechanisms in alcohol, NAFLD and viral-related chronic liver injury, the simplicity of the M1/M2 dichotomy and high degree of macrophage plasticity dependent on the milieu of inflammatory mediators, chemokines and gut-derived microbial products.

The role of macrophages in liver fibrosis is complex. Irrespective of the aetiology, there are common fundamental molecular mechanisms that lead to fibrosis. Mechanistically, persistent or repetitive liver injury results in hepatocyte damage and release of DAMPs which stimulate KC activation. Additionally, KC activation also occurs with the action of PAMPs derived from pathological BT across a dysfunctional gut barrier in cirrhosis, ROS, hypoxia-inducible factor-1α (HIF-1α), increased hepatic concentrations of triglycerides, cholesterol and succinate, and extracellular vesicles (16). Activated KCs produce pro-inflammatory cytokines that contribute to injury (e.g., TNF-α, IL-6 and IL-1β). They also generate, in conjunction with hepatocytes or HSCs, chemokines (e.g., CCL2, CCL5) promoting recruitment of pro-inflammatory, pro-fibrogenic MoMFs (97). Chemokine-driven recruitment may involve different mechanisms; CCL2 in addition to hepatic PC3-secreted microprotein (PSMP) promotes hepatic CCR2+ inflammatory monocyte infiltration and induction of HSCs *via* their CCR2 receptor (98). In NAFLD, murine and human liver biopsies demonstrate CCR2^+^ MoMF accumulation in the portal tracts; KCs do not express CCR2. The marker has diagnostic value with an increase in CCR2^+^ cells observed as fibrosis advances and CCR2^+^ MoMF infiltration throughout the parenchyma in end-stage cirrhosis (78). *In vitro*, viral hepatitis C exposed KCs secrete CCL5 that induces CCR5+ HSC activation through ERK phosphorylation (99). Where there are defined areas of injury, MoMFs form ring-like structures and where damage is more widely distributed, they convene in periportal regions (100, 101). Murine Ly6C^high^ monocytes differentiate into Ly6C^low/+^ macrophages that secrete proinflammatory cytokines and generate ROS. These inflammatory macrophages activate HSCs, generate TNF-α and IL-1β, that promotes HSC survival, and IL-6 that leads to HSC proliferation and production of TIMP1 (102). Other HSC activation routes occur *via* paracrine mechanisms involving JAK2 signalling pathways, NADPH complexes (NOX) and hepatocyte mitochondria-derived DAMPs (16, 103–106). Moreover, these MoMFs release profibrotic mediators such TGF-β, platelet-derived growth factor (PDGF), connective tissue growth factor (CTGF) and TIMPs, which promote myofibroblast ECM production (16, 58).

Not all hepatic macrophages in the setting of chronic liver injury are profibrotic. MoMFs can also exert anti-fibrogenic functions, depending on their differentiation status and the disease stage. This has been shown in murine studies were the deletion of infiltrating MoMFs during fibrogenesis resulted in reduced HSC activation and ECM deposition whilst deletion of MoMFs during the regression stage impaired ECM degradation, thereby exacerbating fibrosis (107, 108). In humans, a distinct population of TREM2^+^CD9^+^ scar-associated macrophages (SAMs) have been observed to occupy fibrotic niches in livers of cirrhotic patients. SAMs are derived from recruitment and differentiation of circulating monocytes, and are expanded early in the disease course (53, 108). Originating from initially pro-inflammatory TGF-β and PDGF-producing infiltrating macrophages, SAMs evolve after a phenotypic switch in the restoration phase of tissue inflammation and function to resolve fibrosis, mainly through the expression of MMPs, including MMP-13, and mediated by macrophage migration inhibitory factor (MIF) (109, 110). TREM2^+^CD9^+^ macrophages have also been reported in NAFLD/NASH models, however, *Trem2* and *Cd9* are not restricted to non-KCs in mice (49, 89, 91, 111). The multiple mechanisms of fibrosis progression and resolution and the co-existence of various macrophage subsets with potentially distinct functions present several opportunities for drug therapies to reverse or ameliorate fibrosis in chronic liver diseases.

### C. Adipose Tissue Macrophages

NAFLD is nowadays one of the most common CLD that is strongly associated with obesity and the metabolic syndrome, and similar to these conditions, its incidence and prevalence are worryingly increasing (91, 92). This disease is underpinned by a pathophysiology that links multiple processes including chronic low-grade inflammation, lipotoxicity, dysregulated bile acid metabolism and altered gut microbiome with enhanced BT. Adipose tissue macrophages (ATMs) are spread throughout the tissue; although their origin under homeostatic conditions is not clear, recent murine fate mapping studies have revealed that resident ATMs can derive from both yolk-sac and bone marrow (monocyte) progenitors (91, 112). How similar these two resident ATM subsets are, it remains to be further investigated. In obese adipose tissue, the size and number of adipocytes increase, to compensate for excess lipid availability. However this containment mechanism may fail, and lead to tissue dysfunction, dyslipidemia and insulin resistance (91, 92). Dying adipocytes can release various triggers (e.g., toxic lipids, adipokines) or DAMPs which create a complex local microenvironment that causes ATM activation. In response, the number of ATMs is markedly increased, with many of them surrounding necrotic adipocytes within crown-like structures, in both mice and humans, an optimal location enabling them to engulf cell debris (91, 92).

Notably, ATMs have been associated with the severity and progression of NAFLD (113). In both mice and humans, the recruitment of macrophages in adipose tissue compartment has been linked with development of insulin resistance and steatohepatitis while ablation of ATMs or surgical removal of adipose tissue in mice normalized insulin sensitivity and partially reversed liver inflammation (92). ATMs secrete inflammatory cytokines such as TNF-α, IL-1β, IL-6 and monocyte-chemoattractant protein-1 (MCP-1) which promote insulin resistance, lipolysis and hepatic lipid flux and accumulation (114). Chemokine axes such as CCL2-CCR2 and CCL5-CCR5 drive further recruitment to adipose tissue, and in part provides the rationale for their dual CCR2-CCR5 antagonism as a therapeutic approach to resolve inflammation and prevent fibrosis progression in NAFLD (75). The phenotype of ATMs does not conform to the dichotomous M1/2 polarisation as once thought, but instead has a unique blend termed a metabolically activated type with distinctive transcriptional profiles that can be influenced by the local adipose tissue microenvironment. Pro-inflammatory macrophage programming may be primed by LPS (perhaps more so with pathological BT), IFN-γ, HIF-1α and saturated fatty acids. Independent of LPS, fatty acids may be taken up *via* the macrophage scavenger receptor 1 (MSR1) with subsequent JNK signalling and inflammation (115). On the contrary, HIF-2α, PPAR-γ and unsaturated FAs exert an anti-inflammatory effect (91, 92, 116). Few groups have recently examined the nature of ATMs in obese adipose tissue by scRNA-seq (112, 117, 118). It has now become evident that ATMs display significant heterogeneity, with various subsets (clusters) identified that may contribute differently to the disease pathology; their specific functions need to be studied in the coming years (91).

### D. Peritoneal Macrophages

The development of ascites, the pathological accumulation of fluid within the peritoneal cavity, is a defining feature of decompensated cirrhosis. Pathogens and bacterial products are readily absorbed by the peritoneal cavity and invoke an inflammatory reaction. This can give rise to SBP which occurs in 10% of hospitalised patients and once established can worsen prognosis including the development of multi-organ failure and increased mortality. SBP is defined by an elevated ascites polymorphonuclear cell count of >250/mm^3^ with or without the positive culture of microbes. In cirrhosis, the pathological BT theory implies a higher exposure to pathogenic material in the peritoneum even in steady state.

Under normal conditions, peritoneal macrophages (PMs) comprise 50-90% of the peritoneal leucocytes and are primarily responsible for clearing debris and pathogens. In both sterile and pathogen-associated injury, interferon-γ primed macrophages orchestrate the immune response, highly expressing MHC class II molecules, generating cytokines and chemokines and exerting strong antimicrobial effector mechanisms (119, 120). Similar to the dichotomy of KC and MoMF in the liver, murine PMs can be subdivided into self-renewing resident macrophages and MoMFs which slowly replace the resident population and acquire a differentiated phenotype (121). Resident PMs have a unique profile including high expression of genes encoding phagocytic receptors (e.g., *Vsig4, Timd4, and Marco*) (122).

In humans, two studies from the same group, one evaluating ascites of patients with cirrhosis and the other abdominal washouts from women undergoing gynaecological abdominal surgery, identified that PMs are distributed in three subpopulations: a) classical-like CD14^++^CD16^-^, b) an intermediate CD14^++^CD16^+^, and c) a large granular CD14^high^CD16^high^ subset. The latter has no corresponding blood monocyte subpopulation (123, 124). The expression of CD14 (LPS related receptor) and CD16 (phagocytic Fcγ receptor) is increased in PMs, compared to circulating monocytes, suggesting that PMs are primed for pathogen defence. Moreover, in patients with cirrhosis and ascites, the CD14^high^CD16^high^ peritoneal macrophage subset represented approximately 30% of total CD14+ cells, compared with approximately 15% in women undergoing gynaecological surgery, perhaps reflective of the enhanced BT in cirrhosis.

Human PMs were also found to have higher expression of other phagocytic markers (e.g., CD11c), cytokine receptors (e.g., CD116 and CD119), antigen-presentation markers (e.g., HLA-DR) and co-stimulatory molecules (e.g., CD40 and CD80) when compared with blood monocytes. The CD14^high^CD16^high^ population was felt to represent an M2-polarised human resident peritoneal macrophage population, as suggested by high GATA-6 expression (similar to mice) (121, 122), enhanced expression of mannose receptor (MR) CD206 and HLA-DR, and higher frequency of IL-10 positive cells. Despite this, they seem to have an immunologically-primed status with increased phosphorylation of ERK1/2, PKB and c-Jun, sensitivity to LPS, robust oxidative burst activity, increased expression of cytosolic dsDNA sensor absent in melanoma 2 (AIM2) and AIM2 ligand-induced mature IL-1β and IL-18 production suggesting they are readied for antimicrobial defence (124–127). Immune priming may vary according to cirrhosis aetiology, with alcohol-related cirrhosis displaying a more pro-inflammatory profile, compared to viral hepatitis C, with higher ascitic IL-12 and lower IL-10 levels (128). Genetic polymorphisms in the *TRAF6* gene coding for the adaptor protein in TLR signalling cascades, confers a less pro-inflammatory state of PMs in cirrhosis and increased risk of SBP (129).

Irvine et al. went further to segregate human PMs by investigating the expression of CCR2 and complement receptor for immunoglobulin (CRIg) which is encoded by the *VSIG4* gene (130, 131). The investigators found a distinct human peritoneal macrophage population that was CRIg^high^CCR2^low^. Compared with CRIg^low^, the CRIg^high^ macrophages were larger, more granular, had higher expression of CD14, CD16, HLA-DR, CD169 and CD163, similar to the aforementioned CD14^high^CD16^high^ resident subset. CRIg^high^ macrophages were characterized by increased gene transcription of efferocytosis receptors (e.g., *MERTK* and *TIMD4*), and showed enhanced phagocytic and microbicidal functions. Of note, high proportions of CRIg^high^ macrophages were associated with reduced liver disease severity (MELD score) and lower proportions were observed during clinical events (e.g., SBP, death) (130).

Stengel and Quickert et al., also identified a distinct subset of CD206^+^CCR2^-^ large peritoneal macrophages (LPM) (in addition to being CD16+, CD163+, CRIg+ and MerTK+) in patients with cirrhosis which were transcriptionally and functionally distinct. When activated, this subset of CD206+ LPMs were more likely to produce pro-inflammatory cytokines, showed resistance to endotoxin tolerance and cleavage of surface CD206. Concentrations of the CD206 soluble form (sCD206) in the ascitic fluid were elevated in SBP, compared to non-infected ascites, reflective of PM activation; high sCD206 ascites levels were predictive of higher 90-day mortality (132). This is in contrast to the historically perceived anti-inflammatory nature of CD206+ macrophages, illustrating their plasticity (133).

Considering the entire human peritoneal macrophage population, the co-expression of M1 and M2 markers points to a constitutive plasticity, responsive to sterile and pathogenic stimuli and programmed to subsequently restore tissue homeostasis (134). The ascitic microenvironment may be crucial in altering the macrophage phenotype and function to suit the insult and promote recruitment of circulating myeloid cells. Hypoxia promotes peritoneal macrophage generation of VEGF and ADM *via* enhanced HIF-1α transcription (135, 136). In the setting of ACLF where cardiorespiratory organ failure might result in hypoxia, the production of vasodilator mediators in such circumstances serves as a mechanism to further worsen circulatory dysfunction. In the setting of pathological BT, high ascites bacterial DNA concentrations, even in the absence of a clinical diagnosis of SBP, are associated with increased proinflammatory cytokine production, increased inducible nitric oxide synthetase (iNOS) expression and subsequent nitric oxide (NO) overproduction, reduced CD14+ peritoneal macrophage HLA-DR expression and poor clinical outcomes (e.g. death and hospital readmission) (137–139).

Infection (SBP), bacteria or bacterial products (e.g., LPS), result in production of inflammatory cytokines such as TNF-α, IL-6, calprotectin and macrophage inflammatory protein type 1 beta (MIP-1β), which promote neutrophil and monocyte recruitment (140, 141). Counter-regulatory mechanisms, to prevent over-exuberant inflammation, result in generation of ascitic IL-10, resistin and reduction in peritoneal macrophage surface expression of CD14, CD16, HLA-DR, CD86 and CD206, which reverse on antibiotic treatment. Reduction in macrophage LPS-receptor CD14 in SBP was associated with impaired phagocytosis (142, 143). Interestingly, Wang et al. using the thermal and CCl4-induced acute liver injury murine models and intravital imaging demonstrated that peritoneal cavity (GATA6+) resident macrophages have the ability to migrate *via* non-vascular routes to the injured liver (144). In the damaged sites, GATA6+ macrophages could be triggered by DAMPs, such as ATP, to exhibit a more alternatively activated state and mediate cell debris clearance, thus aiding tissue repair and revascularisation. Future work will examine if these cells are present in humans and to what extent they modify other liver inflammatory diseases.

### E. Intestinal, Lymph Node and Splenic Macrophages

Beyond the liver, peritoneum and adipose tissue, there is little known regarding compartmental tissue macrophage phenotype and function during chronic liver failure. In health, intestinal macrophages represent a small proportion of the lamina propria immune cells, which are CD33+CD14- and exhibit anergy, hyporesponsive to LPS (145, 146). In cirrhosis, substantial evidence points to a failure of the gut barrier, permitting increased intestinal permeability and BT with increased circulating loads of bacterial DNA and other microbial derivatives, and an increased risk of infection (147). One study identified an activated CD14+TREM1+ intestinal macrophage phenotype in cirrhosis, with high expression of iNOS even in early compensated disease and secretion of NO (148). Such activated macrophages are known to be responsive to microbial challenge, with pro-inflammatory cytokine output (e.g. IL-23, TNF-α and IL-6), and may contribute to the observed intestinal defects (149). Immune defects permitting BT were also observed in mesenteric lymph nodes from patients with decompensated cirrhosis, where circulation-derived subcapsular sinus and medullary cord macrophages expressed the immune-suppressive marker MERTK, compared to non-cirrhotic controls (42). In patients with cirrhosis and portal hypertension, the number and phagocytic activity of splenic macrophages in the red pulp and marginal zone is enhanced, resulting in hypersplenism and cytopenias (150). Enhanced phagocytosis may be explained by the upregulation of micro-RNA miR-615-3p and its action on a ligand-dependent nuclear receptor corepressor (LCoR)-PPARγ axis (151). Finally, there is evidence of spleen-liver organ crosstalk, with splenectomy reversing the M1-dominant hepatic macrophage phenotype in fibrotic livers, ameliorating collagen deposition and regressing fibrosis (152). This is attributed to the disruption of splenic macrophage promotion of hepatic macrophage CCL2 expression, subsequent circulating monocyte liver recruitment and adoption of an M1 phenotype causing further injury and fibrosis (153).

## Macrophage-Related Biomarkers in Liver Disease

Macrophages play a significant role in the development and progression of chronic liver failure. Two scavenger receptors that are highly, but not exclusively, expressed by blood monocytes and macrophages are CD163 and CD206, also known as the haemoglobin-haptoglobin receptor and mannose receptor (MR), respectively (154, 155). The soluble forms of CD163 (sCD163) and MR (sMR), that are present in plasma and body fluids, have been thoroughly investigated as macrophage-related biomarkers in liver diseases (154, 155). Over recent years, numerous studies on sCD163 and sMR have detected increased levels in relation to severity and prognosis in both acute and chronic liver diseases (154, 155). For instance, this has been demonstrated in NAFLD/NASH, viral hepatitis (e.g., HBV, HCV), autoimmune hepatitis, acute liver failure (ALF) and alcohol-related liver disease (132, 156–163). In patients with cirrhosis, sCD163 and sMR significantly correlate with severity (e.g. MELD and Child-Pugh scores) as well as the degree of portal hypertension (164–167); high plasma concentrations of sCD163 associate with variceal bleeding in cirrhotic patients and predict mortality in alcoholic hepatitis (165, 168). Bruns and colleagues recently measured sMR levels in ascites fluid from cirrhotic patients with SBP and found that its concentrations serve as a marker of peritoneal macrophage activation, inflammation and predict 90-day survival (132).

Overall, the highest sCD163 and sMR levels are detected in patients with the most severe forms of liver injury such as ALF, alcoholic hepatitis and ACLF (154, 155). In ACLF, sCD163 and sMR are independently associated with disease severity and prognosis while supplementation of these macrophage biomarkers to standard clinical scores (e.g., CLIF-C ACLF, CLIF-C AD) improved their prognostic performance (158). This accumulating clinical evidence shows robust association of macrophage-related markers with inflammation in liver pathologies and emphasizes the role of macrophages and infiltrating monocytes in liver disease development and progression. Future work will establish the incorporation of these biomarkers in current clinical practice scoring tools to improve prognosis.

## Macrophage-Targeted Therapeutic Approaches in Liver Disease

There is a major unmet need for effective therapies in chronic liver failure and this is of high clinical relevance considering the escalating disease prevalence. Macrophages are central to the pathogenesis of liver diseases as they are involved in the initiation, progression and regression of tissue injury. Macrophages play a key role in liver homeostasis and are among the first responders to an infectious or tissue-damaging insult exerting dual, inflammatory and anti-inflammatory/restorative, functions in the liver. Therefore, hepatic macrophages are an attractive target for developing new therapeutic approaches. Most macrophage-based strategies have been investigated in animal models while some have been evaluated in clinical trials [reviewed in (10, 16, 56, 92)].

**Figure 2:**
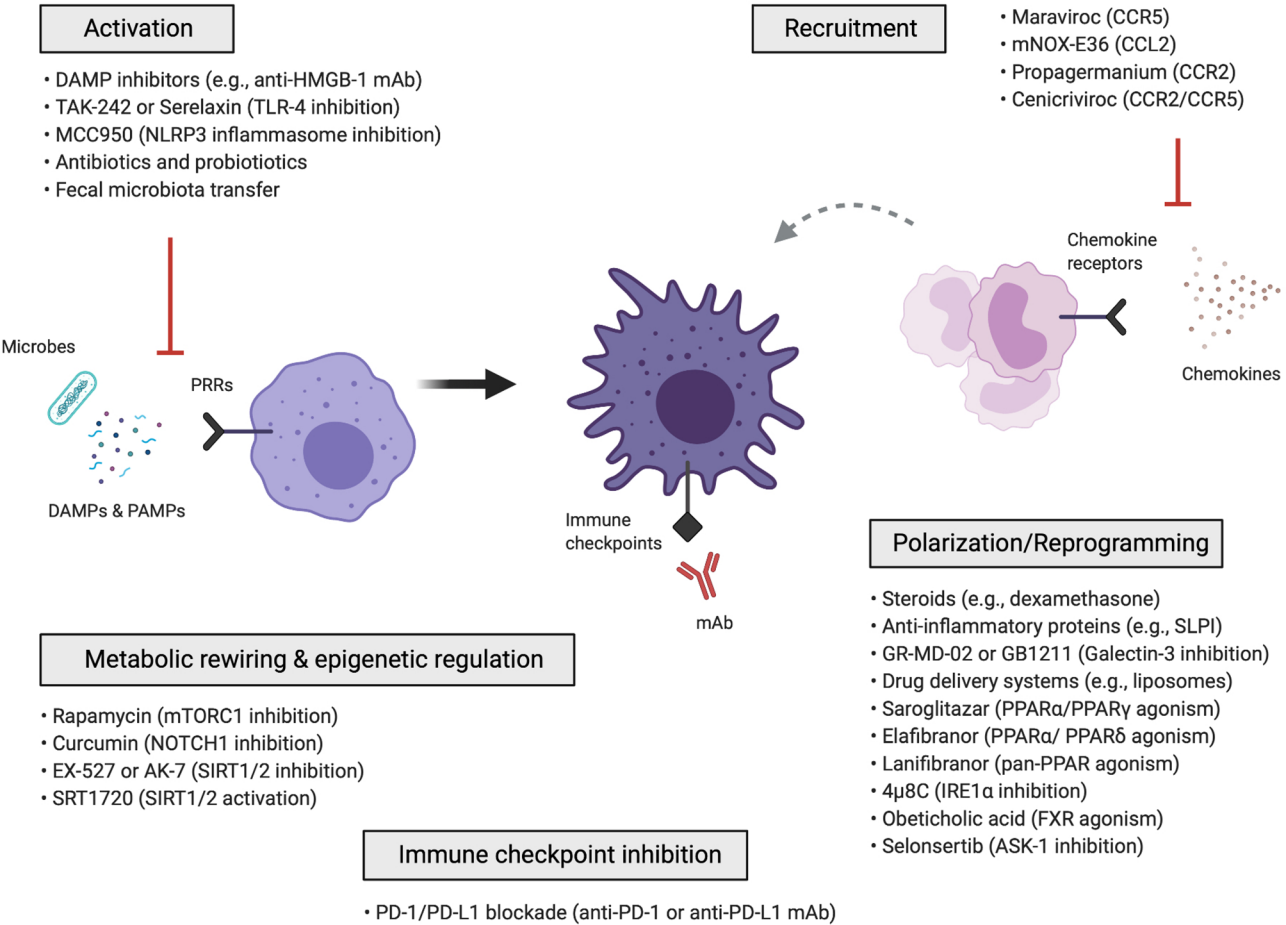
Macrophage-targeted therapeutic approaches for the treatment of liver disease. Schematic overview of the different macrophage-directed therapeutic approaches that can be summarized into those that: (i) inhibit the activation of macrophages, (ii) inhibit the recruitment of monocytes and monocyte-derived macrophages, (iii) reprogram macrophages through anti-inflammatory and polarization mediators, signalling pathways, drug delivery nanosystems, iv) metabolically rewire or epigenetically regulate macrophages, v) modulate macrophages *via* immune checkpoint inhibition. ASK-1, apoptosis signal-regulating kinase 1; CCL, C-C chemokine ligand; CCR, C-C chemokine receptor; DAMP, damage-associated molecular pattern; FXR, farnesoid X receptor; IRE1α, inositol-requiring enzyme 1α; mAb, monoclonal antibody; PAMP, pathogen-associated molecular pattern; PD-1, Programmed-cell-death-1; PD-L1, Programmed-cell-death-ligand-1; PPAR, peroxisome proliferator-activated receptor; PRR, pattern recognition receptor; SIRT1/2, sirtuin-1 and sirtuin-2; SLPI, secretory leukocyte protease inhibitor; TLR, toll-like receptor.

Macrophage-targeted approaches can be categorized into those that: (i) inhibit macrophage activation, (ii) inhibit the recruitment of monocytes and monocyte-derived macrophages, (iii) reprogram macrophages *via* anti-inflammatory polarization mediators and signalling pathways, drug delivery nanosystems, metabolic rewiring, epigenetic mechanisms or immune checkpoint blockade (**Figure 2**). Cell-based therapies using autologous macrophage infusions have also been tested in patients with liver cirrhosis (169); the rationale and potential of utilizing macrophages as agents for cell therapy are reviewed in (57, 170). Here, we summarize the various approaches that have been explored for therapeutic targeting of macrophages in liver diseases.

### A. Inhibition of Macrophage Activation

Changes in gut microbiota composition, increased intestinal permeability and pathological BT into the liver are characteristics of progressive chronic liver disease (e.g, cirrhosis) causing an increase in hepatic levels of endotoxin (e.g., LPS) (8, 35). These gut-derived PAMPs together with liver-derived DAMPs (e.g., HMGB-1, histones) can potentially activate macrophages *via* pattern recognition receptor (PRR) recognition, triggering inflammatory cascades whose activation can be modulated by several approaches. Among those PPRs, the importance of TLR4 is well documented; indeed, TLR4 inhibition (TAK-242 or Serelaxin) has been shown to ameliorate injury and inflammation in rodent models of liver fibrosis, cirrhosis and ACLF (171–174). In the same context, NLRP3 inflammasome inhibition (MCC950) reduces liver inflammation and fibrosis in experimental NASH (175). Finally, PAMP-mediated macrophage activation can be prevented with restoration of normal gut microbiome using broad-spectrum antibiotics, probiotics and fecal microbiota transfer [further discussed in (8, 16, 176)].

### B. Inhibition of Monocyte/Macrophage Recruitment

Tissue-infiltrating monocytes and monocyte-derived macrophages can amplify and perpetuate liver inflammation. Their recruitment into the liver is driven by the chemoattractant properties of several chemokines secreted from activated liver cells (e.g., KCs, LSECs) which interact with chemokine receptors expressed on immune cells. The CCL2/CCR2, CCL5/CCR5 and CCL1/CCR8 are very common chemoattractant axes in liver diseases (16, 56). Chemokine signalling inhibition therefore represents an interesting therapeutic strategy to reduce monocyte recruitment and has proven efficacious in experimental disease models. This can be achieved using monoclonal antibodies against chemokines or chemokine receptors, receptor antagonists, aptamer molecules or small-molecule inhibitors (16, 56).

Interference with chemokine pathways to restrict the influx of inflammatory monocytes is one of the most advanced treatments in NASH-related liver fibrosis. Cenicriviroc (CVC; a dual CCR2/CCR5 inhibitor) efficiently blocks CCL2 mediated monocyte recruitment and has been shown to exert anti-inflammatory and anti-fibrotic effects in various experimental liver disease models (78, 177, 178). These results led to human studies evaluating CVC in NASH patients with fibrosis. Following one year of CVC treatment, a significant number of NASH patients showed good response to the treatment and significant improvement in the histological stage of fibrosis (179). These positive effects were maintained in responders in the second year of CVC treatment (180) and a phase 3 clinical trial is assessing its efficacy and safety (NCT03028740). Other molecules inhibiting cell recruitment include propagermanium (CCR2 inhibitor) (181), mNOX-E36 (RNA-aptamer molecule that inhibits CCL2) (182), maraviroc (CCL5/RANTES inhibitor) (183) and small-molecule antagonist against the G protein-coupled receptor 84 (GPR84) (184) which ameliorate disease in experimental NASH.

Recent scRNA-seq studies have described the macrophage heterogeneity in murine NASH, consistently demonstrating that resident KCs (ResKCs) are lost during NASH progression and recruited monocytes enter the liver and become monocyte-derived KCs (MoKCs) or temporary MoMFs (49, 87–89). The distinct roles in disease pathology, functions and relationship between these subsets remain to be explored. For instance, what triggers ResKC death and why MoMFs are protected from this? Furthermore, it’s not clear whether the strategy to reduce monocyte recruitment in NASH *via* CCR2/CCL2 inhibition could affect both MoKCs and recruited MoMFs, or the latter cells only. Of note, NASH-related liver fibrosis was increased in *Ccr2* KO mice (89). Could the timing of CCR2 blockade influence macrophage composition and also alter tissue remodelling? Differences between murine and human macrophage subset functions are likely. Further exploration and better understanding of macrophage heterogeneity in NASH will inform us how to specifically target these critical immune cell subpopulations.

### C. Macrophage Reprogramming

Hepatic macrophages exhibit great functional plasticity. Therefore, a key therapeutic approach is to induce a switch from an inflammatory to an anti-inflammatory/restorative type, aimed to promote resolution of inflammation and accelerate tissue regeneration. Such macrophage reprogramming can be achieved using anti-inflammatory mediators including steroids (e.g., dexamethasone), prostaglandin E2 (PGE_2_), macrophage colony-stimulating-factor-1 (CSF1) and secretory leukocyte protease inhibitor (SLPI) (10, 43, 185). Galectin-3, mainly expressed in macrophages, is shown to exert inflammatory functions and profibrogenic effects on hepatic stellate cells (186). Despite showing promising results in rodent models, the galectin-3 inhibitor belapectin (GR-MD-02) did not alleviate fibrosis in a phase 2 trial in patients with NASH with cirrhosis and portal hypertension (187, 188). A study evaluating the efficacy and safety of another galectin-3 inhibitor, GB1211, in NASH patients is currently ongoing (NCT03809052). Other targets explored aiming to prevent macrophage activation and promote M2-like polarization include inositol-requiring enzyme 1α (IRE1α) (189), farnesoid X receptor (FXR) (190), apoptosis signal-regulating kinase 1 (ASK-1) (191) and obeticholic acid which is a strong FXR agonist with promising results from an early phase clinical trial in NASH (192).

Due to the anatomical location (liver sinusoids) and high scavenging capacity (e.g., mannose receptor) of KCs, the systemic administration of different drug delivery nanosystems (e.g., polymers, liposomes and microbubbles) leads to their accumulation in the liver (193, 194). This highlights their potential for developing new hepatic macrophage-targeted therapies. For instance, the administration of dexamethasone-loaded liposomes reduced liver inflammation in murine models of acute hepatitis and CCL_4_-induced chronic toxic liver injury, and this was associated with reduced number of hepatic T cells and induction of an M2-like/restorative macrophage phenotype (195). While such drug carrier materials target hepatic myeloid cells, liver fibrosis also affects their targeting efficiency, supporting the need to adapt nanomedicine-based approaches in chronic liver disease (194, 196).

Another strategy to induce anti-inflammatory macrophage polarization and consequently also ameliorate disease progression is by promoting signalling through the PPAR pathways. PPARs are nuclear transcription factors with multiple functions in NASH pathology, affecting inflammation, lipid and glucose metabolism (197). Recent work has shown that *in vivo* treatment with saroglitazar, a PPARα/PPARγ agonist, is associated with reduced inflammation and regression of fibrosis in experimental NASH models (198). In a human study, elafibranor, a PPARα/PPARδ agonist, was shown to attenuate liver inflammation without fibrosis worsening in NASH patients (199). Furthermore, *in vitro* treatment with lanifibranor, a pan-PPAR agonist, reduces the expression of inflammatory genes in murine macrophages and patient-derived circulating monocytes with palmitic acid and increases the expression of lipid metabolism related genes. The anti-inflammatory actions of lanifibranor can be induced through PPARδ agonism as demonstrated by evaluating the effects of individual PPAR agonists (200). Moreover, lanifibranor treatment inhibits hepatic MoMF accumulation, one of the key events preceding fibrosis (78). Lanifibranor is currently investigated in a phase 2 trial in NASH patients (NCT03008070).

Recently, LC3-associated phagocytosis (LAP), a non-canonical form of autophagy that shifts monocyte/macrophage phenotype to an anti-inflammatory type, was reported to be a protective mechanism against fibrosis and systemic inflammation in cirrhosis (201). LAP is enhanced in peripheral and hepatic monocytes from patients with liver fibrosis or those who progress to cirrhosis. Pharmacological inhibition of LAP in patient-derived monocytes or LAP genetic disruption in mice exacerbated inflammation and fibrosis after CCl_4_-induced liver injury whereas enhancing LAP reduced inflammation and liver fibrosis (201). Moreover, activation of LAP is lost in monocytes from ACLF patients and can be restored by targeting this pathway (201). This suggests that sustaining LAP may open new therapeutic perspectives for patients with chronic liver diseases.

### D. Macrophage Metabolic Rewiring and Epigenetic Regulation

An additional approach could be the metabolic rewiring of hepatic macrophages to modulate their polarization and regulate their function for liver disease treatment (92). Pharmacological promotion of autophagy by targeting a key metabolic regulator, the mammalian target of rapamycin complex 1 (mTORC1), improved high fat diet-induced steatohepatitis in mice by altering lipid metabolism, macrophage polarization, the inflammatory responses and autophagy (202). Interestingly, mice with macrophage-selective mTORC1 ablation displayed an M2-like phenotype, reduced liver inflammation and improved insulin sensitivity (202). Another study has revealed a crucial role for the NOTCH1 pathway in inducing M1-like activation of hepatic macrophages by promoting mitochondrial oxidative phosphorylation and ROS as well as M1-related gene expression (68). Conditional NOTCH1 deficiency in myeloid cells attenuated hepatic macrophage M1-like activation and inflammation in murine alcoholic steatohepatitis and markedly reduced lethality following endotoxin-mediated fulminant hepatitis (68).

The concept of “innate immune memory” has arisen over recent years which may open another window of opportunity for new therapies in chronic liver diseases (203). This idea stems from studies demonstrating that epigenetic mechanisms regulate macrophage function by imprinting them with a “memory response” towards future stimuli. Therefore, macrophages can mount a qualitatively different response, either exaggerated (“trained”) or impaired (“tolerant”), upon exposure to repeated challenge (204, 205). In the context of acute and excessive inflammation, tolerance can act as a protective mechanism to dampen the host’s inflammatory responses to prevent tissue damage. For instance, in response to LPS or pathogen exposure, monocytes and macrophages modify their histone acetylation and methylation traits, dictating gene expression patterns upon subsequent stimulation (206–208). Some of these mechanisms are implicated in rewiring of intracellular metabolic activities affecting the balance between glycolysis and fatty acid oxidation (209, 210). For example, inhibitors of histone deacetylases sirtuin-1 and sirtuin-2 (SIRT1/2) have shown the capacity to reverse immune paresis in experimental murine sepsis (211, 212). Whether such a molecular memory imprinting of anti-inflammatory monocytes and macrophages could be achieved in chronic liver failure is worth exploration.

### E. Macrophage Regulation *via* Immune Checkpoint Blockade

Immune checkpoints constitute a complex array of receptors (e.g., PD-1, CTLA-4) and their ligands (e.g., PD-L1/PD-L2, CD80/C86) expressed on both innate and adaptive immune cells, which exert key regulatory roles during homeostasis and inflammatory pathologies, mainly in chronic infection, sepsis and cancer. Immune checkpoint inhibition has become an emerging therapeutic strategy in various liver diseases (203, 213). For instance, we have demonstrated that peripheral CD4+ T cells from ALF patients have increased CTLA-4 expression and reduced proliferative response to stimulation that can be enhanced *via* CTLA-4 inhibition *in vitro* (214). Furthermore, defects in adaptive and humoral immunity can be partially rescued by *in vitro* PD-1 blockade in patients with alcohol-related liver disease and viral hepatitis B infection (215, 216). Importantly, the therapeutic reversal of immune exhaustion using immune checkpoint monoclonal antibodies (mAb), alone or in combination with other drugs, has been shown to be effective and with good safety profiles in hepatocellular carcinoma (217–219).

Most checkpoint pathways have been first described as regulators of T cell immunity, but it is now clear that their effects are not limited to T cells only. For instance, accumulating evidence has revealed an crucial role for PD-1/PD-L1 signalling on altering myeloid cell function in sepsis and cancer (220–222). PD-1 and PD-L1 can be induced on monocytes and macrophages through TLR ligands (e.g., LPS) and cytokines (e.g., TNF-α, IL-6 and IL-10). Interestingly, PD-1 and PD-L1 monocyte expression is associated with increased mortality in septic patients while PD-1/PD-L1 blockade restores innate responses in experimental sepsis (223–226).

Many features of sepsis resemble those observed in acute (e.g., acetaminophen overdose) or chronic (e.g., decompensated cirrhosis, alcohol-related ACLF) liver failure patients who often acquire bacterial infections (10, 203, 213). We recently explored the PD-1/PD-L1 pathway in acetaminophen-induced ALF (227). During resolution of murine liver injury, impaired hepatic bacterial clearance and increased PD-1 and PD-L1 expression of KCs and lymphocyte subsets were detected. Compared to wild-type, PD-1 deficient or anti-PD-1 mAb treated mice with liver injury showed improved KC bacterial clearance, reduced bacterial load and protection from sepsis (227). We also found up-regulated PD-1 and PD-L1 expression of peripheral monocytes and lymphocytes in patients with ALF and increased plasma soluble PD-L1 levels predicting mortality and development of sepsis (227). Similarly, another study demonstrated overexpression of PD-L1 in peripheral monocytes and liver macrophages in cirrhotic patients who displayed impaired hepatic bacterial uptake (228). Interestingly, monocyte PD-L1 was associated with disease severity and infection risk in cirrhosis while anti-PD-L1 mAb treatment restored the phagocytic capacity of macrophages and reduced bacterial dissemination in mice with chronic liver injury (228). These studies indicate that immune checkpoint blockade may be an effective and safe strategy for restoration of defective antibacterial responses in chronic liver failure patients, where conventional treatment options are currently very limited.

## Conclusions

In conclusion, intensive research over recent years has significantly improved our knowledge on macrophage diversity and plasticity in the context of liver diseases. Technical advances in experimental models and tools, such as single-cell approaches, have enabled us to dissect the key cellular and molecular functions of tissue macrophages, uncovering critical pathophysiological changes involved in different disease states such as liver fibrosis. This forms the basis for future studies that will further deepen our understanding of liver disease pathogenesis, thus enabling the identification of novel immunotherapeutic approaches for patients across the spectrum of chronic liver disease.

## Author Contributions

All authors listed have made a substantial, direct, and intellectual contribution to the work and approved it for publication.

## Funding

ET has received funding support from Imperial College London (Imperial College Research Fellowship award) and funding support from the Rosetrees Trust (CF2\100002).

## Conflict of Interest

The authors declare that the research was conducted in the absence of any commercial or financial relationships that could be construed as a potential conflict of interest.
